# Trauma-Informed Undergraduate Medical Education: A Pathway to Flourishing with Adversity by Enhancing Psychological Safety

**DOI:** 10.5334/pme.1173

**Published:** 2024-06-03

**Authors:** Robert C. Whitaker, Georgia B. Payne, Maeve A. O’Neill, Megan M. Brennan, Allison N. Herman, Tracy Dearth-Wesley, Henry F.C. Weil

**Affiliations:** 1 Professor of Clinical Pediatrics, Columbia University Vagelos College of Physicians and Surgeons, New York, NY, US; 2 Director of Research and Research Education in the Columbia-Bassett Program, Columbia University Vagelos College of Physicians and Surgeons, New York, NY, US; 3 Bassett Medical Center and Bassett Research Institute, Cooperstown, NY, US; 4 Second year medical student in the Columbia-Bassett Program, Columbia University Vagelos College of Physicians and Surgeons, New York, NY, US; 5 Assistant Professor of Clinical Psychiatry, Columbia University Vagelos College of Physicians and Surgeons, New York, NY, US; 6 Assistant Clinical Professor of Psychiatry, Columbia University Vagelos College of Physicians and Surgeons, New York, NY, US; 7 Research Associate in the Columbia-Bassett Program, Columbia University Vagelos College of Physicians and Surgeons, New York, NY, US; 8 Senior Research Associate in the Columbia-Bassett Program, Columbia University Vagelos College of Physicians and Surgeons, New York, NY, US; 9 Professor of Clinical Medicine, Columbia University Vagelos College of Physicians and Surgeons, New York, NY, US; 10 Senior Associate Dean for the Columbia-Bassett Program, Columbia University Vagelos College of Physicians and Surgeons, New York, NY, US and Bassett Medical Center and Bassett Research Institute, Cooperstown, NY, US; 11 Chief Operating Officer of Bassett Medical Center, Cooperstown, NY, US; 12 Chief Clinical Officer and Chief Academic Officer of Bassett Healthcare Network, Cooperstown, NY, US

## Abstract

We describe the Life Experiences Curriculum (LEC), which attempts to integrate medical student well-being with trauma-informed medical education. The long-term goal of LEC is to help medical students flourish with adversity and trauma, where flourishing refers to having a sense of purpose that arises from awareness of one’s strengths and limitations, shaped by life experiences. The short-term goal of LEC is to develop students’ relational capacities, such as acceptance and awareness of self and others, while building and maintaining students’ psychological safety. We describe the conceptual rationale for these goals and the curriculum’s development, implementation, evaluation, and limitations. The curriculum extends over four years and involves a preclinical seminar and students’ individual and group reflection sessions with LEC faculty. The seminar addresses the coexistence of trauma and flourishing across life experiences, as well as how safety in relationships is impaired by traumatic experiences and must be restored for healing and growth. The physician faculty have no role in student evaluation and co-lead all LEC activities. LEC is intended to provide students with new language for understanding the process of trauma and flourishing in both individuals and systems and to build and sustain students’ relational capacities. There are ongoing efforts to re-imagine self-care as communal-care in which care and support are given and received in a community of students and faculty. Such a model may help build the relational capacities needed to deliver trauma-informed care and also promote flourishing with adversity in healers and in those seeking to be healed.

## Background and Need for Innovation

There is emerging consensus that patients would benefit if physicians and healthcare systems had greater sensitivity about how adverse life experiences may affect patients’ health and responses to care [1]. As a result, medical educators are developing curricula for trauma-informed medical education [2,3,4]. Concurrently, there is concern about the prevalence of burnout, suicide, and poor mental health among physicians, including those in training [5,6]. However, medical student well-being is not generally a major goal of the evolving approaches to teaching trauma-informed care (TIC). Furthermore, the content on student well-being appears to emphasize self-care as a set of skills to be developed and practiced by individual students to cope with the stress and demands of medical training, including exposure to vicarious or secondary trauma and moral injury [3] (see Supplement S1: Stress in medical education).

We created the Life Experiences Curriculum (LEC) to integrate the related needs for medical students to learn about TIC and sustain their own well-being. We conceptualized student well-being as flourishing with adversity, and our goal was to help students develop the relational capacities of awareness and acceptance of self and others. We viewed these relational capacities, combined with knowledge about trauma, as necessary for becoming a trauma-responsive physician and, ultimately, for flourishing with adversity. We prioritized students’ psychological safety to foster in them the ability to reflect on both their positive and adverse experiences, integrate their personal and professional development, and pursue their well-being as a collective activity with students and faculty in which they give and receive care. Our purpose is to describe the development and implementation of LEC with a focus on how and why we tried to create psychological safety and help students develop and sustain their relational capacities so they could flourish with adversity.

## Goal of Innovation

The goal of LEC is to help students develop and sustain four relational capacities—self-awareness, other-awareness, self-acceptance, and other-acceptance, which can be thought of, respectively, as mindfulness, empathy, compassion for self, and compassion for others [7]. Psychoeducation about trauma is a core part of LEC, but we felt students needed to integrate that knowledge with these relational capacities in order to recognize and respond to patients who may have experienced trauma and achieve the longer-term goal of flourishing with adversity. In LEC, we here consider flourishing as synonymous with eudaimonic well-being, or having a sense of meaning and purpose achieved through an ongoing process of awareness and acceptance of one’s evolving strengths and limitations [8]. We consider adversity to be events or circumstances that are unwelcome or unwanted. Many cannot easily be anticipated or prevented, and some, but not all, may be traumatic in terms of their lasting impact on functioning [1]. By flourishing we do not mean resilience, which is often characterized as resistance to symptoms of burnout, suicidality, or depression in the face of chronic stress or returning to a baseline of languishing after experiencing such symptoms. Nor do we mean hedonic well-being, characterized by happiness, life satisfaction, or positive affect [9] (see Supplement S2: Conceptual basis of flourishing with adversity as an outcome of LEC).

## Development and Implementation of Innovation

LEC occurs within the Columbia-Bassett Program [10], a track begun by one of us (HW) in 2010 for approximately 10 students per year within Columbia University’s Vagelos College of Physicians and Surgeons in New York, NY, USA. LEC was developed over several years before it was implemented in the 2021–2022 academic year. The development process included the following: 1) a symposium (2015) involving Columbia-Bassett students and nationally-recognized experts in the areas of developmental trauma [11], organizational trauma [12], developmental neurobiology of stress [13], and developmental origins of flourishing [14]; 2) the creation of a research program (2018) involving three of us (RW, AH, and TDW) to inform the development of LEC, which included the formal evaluation (2017–2019) of a course on TIC for early childhood educators [15,16]; 3) training of the LEC co-leaders (RW, MO, and MB) in TIC (2019–2022); and 4) ongoing review of the emerging literature in TIC. In developing LEC, the authors combined their experience in psychiatry, pediatrics, medicine, education, and public health in the realms of teaching, clinical practice, and research (see Supplement S3: Columbia-Bassett Program and development of LEC).

LEC occurs across all four years of medical school and has two main activities—a pre-clinical seminar and reflection sessions. The in-person seminar begins in the first year and meets nine times for 90 minutes over 12 months. All nine seminars (Table 1) are co-led by the three curriculum co-leaders (RW, MB, and MO), who are salaried faculty physicians with more than 30 years of combined experience in clinician educator roles. Before each seminar, students are given reading material that takes about one hour to complete. Reflection sessions include two formats, individual and group, and all occur monthly for 50 minutes via video-conferencing. Individual sessions are 1:1 with co-leaders, rotate across the three co-leaders, and run from the first month of medical school through the end of the major clinical year (MCY), after which they are optional. During the MCY, there are also group reflection sessions that focus on TIC-related topics or clinical experiences brought to each session by the students. Group sessions include all 10 Columbia-Bassett students in their MCY and the co-leaders. Optional group sessions are available each month following their MCY. Students are encouraged to contact any of the co-leaders on an ad hoc basis. Finally, the process of conducting LEC, apart from its content, involves explicit strategies (Table 2) to create and maintain psychological safety (see Supplement S4: Rationale for prioritizing psychological safety).

**Table 1:** Overview of pre-clinical seminar content.


SESSION TITLE		KEY TOPICS	TEACHING GOALS

1.	Trauma-Informed Care	Definition of trauma as an event, experience, and effect Assumptions in a trauma-informed approach: realize, recognize, respond, resist Guiding principles of a trauma-informed approach (per Substance Abuse and Mental Health Services Administration) [1]	Explore the way in which context affects whether adverse events or circumstances are experienced as trauma and how that context may alter one’s perspective on an adversity, over time or relative to another person exposed to a similar adversity Help students understand that all stress and adversity are not necessarily traumatic and may even contribute to growth Recognize that some traumatic events experienced by prior generations can still negatively impact the functioning of subsequent generations, even if they did not experience those events

2.	Nature and Impacts of Trauma	Adverse childhood experiences and realms of trauma Negative health impacts of trauma Responses of medicine and public health	Describe the range of categories of adverse childhood experiences, extending beyond those in the household to include those in the community Discuss the limitations of using those experiences in predicting the future health of individuals, even as they may explain the burden of health problems in populations

3.	Response to Trauma and Healing	Attachment: “operating system” for social functioning Secure base: psychobiological and behavioral safety	Introduce attachment theory, a topic rarely presented in the core medical education curriculum, despite its implications across the lifespan for health, well-being, healing, and growth Review attachment theory’s conceptual basis for the following: 1) early life experiences, even before the establishment of autobiographical memory, can both positively and negatively impact the development of identity and the social nervous system, 2) healthy relationships over time heal trauma, and 3) healthy relationships over time are both a reflection and cause of human flourishing

4.	Application to Caring for Others	Inquiring about people’s life experiences Responding to people’s life experiences	Emphasize the importance of developing and maintaining psychological safety for both one’s self and the patient Introduce the concept of engaged or therapeutic presence, including the therapeutic role of listening, nonverbal language, and the autonomic nervous system in conveying safety and threat

5.	Application to Caring for Self	Sustainable care and compassion How caring for self relates to caring for others	Present caring for oneself as a communal, rather than individual, activity that involves creating a relational field of caring support with faculty and fellow students Discuss learning to receive care and compassion from self and others rather than only giving care and compassion to others Introduce self-compassion and the factors that may block it

6.	Reconnection and Reflection	Applying the Life Experiences Curriculum personally and professionally	(*This session follows the summer break*) Restore community through a group reflection on the experiences of the summer

7.	Dissociation and Re-enactment	Definitions and examples of dissociation and re-enactment Application in care of self and others	Describe dissociation and re-enactment, which are topics that appear to be rarely covered in trauma-informed medical education Discuss how these two phenomena are highly prevalent responses to trauma and can help explain otherwise confusing and enigmatic behaviors that are manifest not only by patients and colleagues but even by organizations Help students understand when and how dissociation may be a necessary and adaptive short-term response to the experience of extreme stress in their work and how it can even contribute to flourishing with adversity

8.	Organizational Trauma	Parallel process: individual and organizational response Communication in trauma-organized systems Decision-making in trauma-organized systems	Explore how systems under high levels of chronic stress, including most healthcare systems, behave in response to that stress Help students recognize and resist traumatization and re-traumatization of patients that can occur because of care providers’ own responses to working in hierarchical, stressed-organized, healthcare systems

9.	Flourishing with Adversity	Moving from trauma to flourishing Relational capacities in community Making others feel safe and seen	Introduce strategies for flourishing with adversity, with an emphasis on building and sustaining relational capacities within a community of care


**Table 2:** Strategies used in LEC to create and maintain psychological safety.


Students are not evaluated in LEC LEC faculty co-leaders have no role in evaluating students outside of LEC LEC faculty co-leaders demonstrate vulnerability by sharing their own life experiences LEC faculty co-leaders use a co-leadership and co-teaching model^a^ LEC faculty co-leaders review guidelines for maintaining psychological safety in the group^b^ – Each person has different ways of reaching psychophysiological safety – Everyone is invited to participate and everyone has the right to pass – Respect is shown for all opinions and perspectives – Air space is fairly shared – Confidentiality is preserved – Question sensitivity is observed^c^ – Side conversations are kept to a minimum – Preparation and engaged presence are practiced^d^


a One of the co-leaders monitors students’ non-verbal communication, including the desire to speak or expressions of distress that might signal the need for support. b Adapted from group discussion guidelines used by Lakeside Global Institute (North Wales, PA) in their trauma training courses. c Avoid asking probing or clarifying questions about the shared experience of another that may be asking them to share more than they are comfortable sharing. d Preparation is both cognitive (conveying respect and interest by reviewing the material for discussion before the seminar) and emotional (being ready for mindful attention to the feelings and thoughts of self and others during the seminar). Engaged presence (discussed more fully in seminar 4 [see Table 1]) reflects “being as doing,” by showing a genuine interest in the experiences of another and affirming the feelings arising from those experiences by listening with openness and sensitivity [15].

### Pre-clinical seminar

The seminar provides students with conceptual frameworks and language that allow them to better understand how life experiences may be related to health, well-being, healing, and growth. Some of those life experiences may be traumatic, but we did not include the word trauma in naming LEC because an understanding of trauma alone would not serve the long-term goal of helping students or their patients flourish with adversity. Across the seminar sessions (Table 1), we present the paradox of how adversity and flourishing can occur in both one’s personal life and in clinical practice and how understanding both positive and negative experiences is a necessary part of being a trauma-responsive physician.

During a mix of large and small group activities, including the discussion of clinical vignettes, students engage in active dialogue, which is enhanced by psychological safety and leads to better integration and retention of the curriculum content. Each session occurs over an evening meal and is organized with a consistent structure that begins with students and co-leaders sharing reflections about their experiences since the prior session. This opportunity familiarizes students with the group reflection process that characterizes LEC during their MCY, allows students to apply the language and concepts they are acquiring during the seminar series, and models and builds relational capacities associated with engaged presence [15] (see footnote d in Table 2). The sessions are spaced monthly to allow the students time for reflection and integration as they accumulate new experiences, especially their early interactions with patients.

### Reflection sessions

The group and individual reflection sessions help students develop the relational capacities they need to flourish with adversity. We believe these capacities are enhanced in the reflection sessions by providing students the affordance to safely reflect on life experiences, both their own and those of others, with attention to how those experiences affect thoughts, feelings, and behaviors [17]. By reflecting on what *has happened*, we hope to increase students’ abilities to reflect on what *is happening* in their interactions, enhancing their abilities to both observe and participate in the moment. The sessions are not “therapy,” even as they might be experienced as therapeutic.

Students furnish the content for the sessions. They are encouraged to bring forth experiences from work or outside work. When caring for patients, students are affected by their own life experiences, especially their relationships with family, friends, or intimate partners. This can occur, for example, when a student, whose parent has an alcohol use disorder, cares for a patient experiencing the health impacts of chronic alcohol use. Caring for that patient could, in turn, also affect that student’s relationship with their parent. The student’s relational dynamics with patients and others may be impacted when they become aware of how patients’ traumatic life experiences impact illness, disease, and healing. This can even affect a student’s compassion towards themselves, their classmates, and those outside of work. When students discuss emotionally challenging interactions or their responses to people from different backgrounds, we try to affirm their responses and model in our reflections with them a position of openness, non-judgment, and curiosity about their response or those of their patients and colleagues. The simultaneous reflection on both work and non-work experiences, generating both positive and negative affect, allows the student to explore how these varied experiences and contexts relate to one another and how they reveal one’s strengths, limitations, and values. In the example above, there might be an opportunity to reflect with the student on why caring for a patient with chronic alcohol use might be challenging and how compassion for self and others are related. We view the act of flourishing with adversity as the aspirational functioning of a trauma-responsive physician, which only occurs when there is an integration, rather than separation, of work and non-work experiences and identities.

Towards this goal of flourishing, we especially encourage reflection on life and training experiences that bring students joy and are perceived by them as fulfilling or empowering. For example, in reflecting with students on how they experience using their gifts, we see an opportunity to help students with the self-realization of their daemon or unique talents [18]. This provides the opportunity to consider with students the different medical specialties or practice settings that they might select in a process of self-actualization.

## Evaluation of Innovation

LEC is being continually modified in response to student feedback, our experience as teachers, and the emerging literature. One modification was to increase psychological safety in the seminar by beginning individual reflection sessions in September of the first year rather than in January, when the pre-clinical seminar began. Another modification was to allow more time in the seminar for the students to reflect on their early clinical encounters, including those in which they witnessed approaches to care that they perceived could have been more trauma-informed or found themselves feeling psychologically unsafe.

The main impact we have observed during implementation is students’ engaged presence in their interactions with us as faculty co-leaders and with one another. As many as half of the students have spontaneously remarked to one of us that LEC has been the most “meaningful” and/or “important” part of their medical education and that it is something that they “look forward to.” Students have a full and demanding curriculum outside of LEC in which they are evaluated, but almost without exception, they all attend the seminar sessions and rarely miss a reflection session. If they need to miss a reflection session because of unforeseen conflict, they take initiative to reschedule.

With regard to formal evaluation, we are continuing to consider the pragmatic constraints in developing a control group (or condition) and to using a random-assignment design. We have implemented LEC with a small number of self-selected students in a single track in a single medical school, and these students matriculate into the track, in part, based on their interest in learning about TIC and having longitudinal relationships with patients and faculty. In addition, there is no existing control curriculum, exposure to LEC cannot be masked, and “spill-over” effects to students not receiving LEC are difficult to prevent or assess. Furthermore, the impacts of the curriculum may only be manifest after many months, even after medical school.

With these caveats in mind, our vision for formally evaluating LEC is based on our prior research with early childhood educators, including a random-assignment evaluation of a TIC curriculum [15,16] and the subsequent development of a self-report measure of relational capacity [7]. When we examined the qualitative outcome assessments in this evaluation with early childhood educators, we found that meaningful impacts had been missed with our quantitative assessments that used validated instruments to assess psychological constructs. Furthermore, we came to understand through our qualitative data that the key relational capacities of awareness and acceptance of self and others may be necessary to flourish with adversity in settings like health care and education, where trauma is ubiquitous. Although any evaluation of LEC would require both qualitative and quantitative methods, we would propose a relational capacity score [7] and a flourishing score [8] as the primary and secondary quantitative outcome measures, respectively (see Table S1).

## Critical Reflection on the Process

LEC would ideally be undertaken alongside organization-wide efforts to increase TIC and reduce the avoidable trauma in medical training [19]. However, that is not the case, and the faculty and residents that the students work with on clinical rotations are largely unaware of LEC. Therefore, students in LEC become familiar with the language, concepts, practices, and approaches to care that may be unfamiliar to some of their clinical preceptors, and students may feel isolated once their perceptions and responses in clinical settings are altered by applying a “trauma-informed lens.” We have been mindful to name and discuss these possible challenges, and the reflection sessions frequently involve conversations about how best to respond in situations in which students observe approaches to care that are not trauma-informed. Although the preclinical seminars appear to build group trust and equip students with new language about the nature of traumatic experiences and human responses to those experiences, we suspect the more time-intensive, longitudinal reflection sessions contribute most to building and sustaining students’ relational capacities. These reflection sessions are much harder to implement on a larger scale because the faculty co-leaders must be trauma-informed, clinically experienced, collaborative, available in a somewhat flexible manner, able to maintain non-evaluative roles, and have authentic generative and nurturing motivations. However, we suspect that with proper institutional support, such faculty could be available and even form a community of care [20] that, along with students, helps medical schools and academic medical centers begin the process of becoming trauma-informed organizations that also flourish with adversity (see Supplement S5: Future directions).

## Conclusion

LEC addresses the related educational needs of medical students to learn about TIC and sustain their well-being. The two components of LEC—a year-long preclinical seminar and four years of longitudinal reflection sessions with non-evaluative faculty co-leaders—aim to help students develop and maintain the relational capacities that support flourishing with adversity. By creating psychological safety for reflection, we help students integrate their personal and professional development and recognize how they, along with their patients and colleagues, are shaped by their life experiences, both positive and traumatic.

## Additional File

The additional file for this article can be found as follows:

10.5334/pme.1173.s1 Supplementary Material. Supplement S1–Supplement S5 and Table S1.
