# Supplementary material for: Trauma-Informed Undergraduate Medical Education: A Pathway to Flourishing with Adversity by Enhancing Psychological Safety
Source: Perspect Med Educ. 2024 Jun 3;13(1):324–31. doi: 10.5334/pme.1173 (PMC11166023; doi:10.5334/pme.1173)
Supplement: Supplementary Material. — Supplement S1–Supplement S5 and Table S1. [file pme-13-1-1173-s1.pdf]

Section S1. Stress in medical education: sources and implications

Medical students often face unrelenting performance pressure and high stakes evaluation inside a strict hierarchy with intense peer competition and long work hours. They also witness ubiquitous inequity within healthcare, have almost daily engagement with patients and colleagues who are suffering, and are known to experience significant moral injury [1, 2]. This constellation of factors in medical education is not new, but we now understand that it can induce in students a state of threat physiology, which limits students' self-regulation, growth, and learning [3]. This constellation of factors can also limit the time or setting for restoration, reflection, and integration of their experiences.

The evidence and impact of this structural adversity in medical education may lead to the poor mental health of medical students [4], which, even when not manifesting as depressive symptoms, may manifest as languishing rather than flourishing [5]. Many medical students are still in a sensitive period of brain development and face the challenges of emerging adulthood, including emancipating from their family of origin, forming identity, creating and sustaining romantic partnerships, and building families of their own [6].

Section S2. Conceptual basis of flourishing with adversity as an outcome of the Life Experiences Curriculum (LEC)

We use the term flourishing to refer specifically to eudaimonic (or psychological) well-being [7-9]. The most distinguishing feature of this multi-dimensional construct is living with a sense of purpose in which one's activities are carried out in accordance with ongoing awareness and acceptance of one's unique abilities (talents or gifts) and limitations [10]. Thus, flourishing reflects the processes of self-realization (understanding who we are) and self-actualization (becoming who we are) [11]. Because this characterization of psychological functioning does not exclude or require the self-appraisals of life satisfaction or happiness, central to hedonic well-being [7, 8], flourishing as eudaimonic wellbeing is compatible with the reality of life's adversities and traumatic experiences [12]. Ryff's Psychological Well-Being Scale [9] is the most widely used measure of flourishing and assesses six dimensions: purpose in life, self-acceptance, positive relations with others, personal growth, environmental mastery, and autonomy (**Table S1**). These dimensions distinguish flourishing with adversity from coping or resilience [13], terms often used to describe physicians who manage to function amidst adversity. Flourishing with adversity is also different from post-traumatic growth [14] because flourishing does not require the adversity to be traumatic.

The goal of flourishing with adversity is well-matched to the circumstances of medical school. Most students arrive with a keen sense of purpose and some awareness of the unavoidable adversity in caring for those facing suffering and loss. In working with patients and in choosing their field of practice, students must identify their gifts and limitations, which are shaped by their own life experiences, including adversities or traumas. At the same time, the system of medical education has avoidable adversities, which can be traumatic insofar as they result in ethical erosion, moral distress, or a sense of loss and betrayal [15-17]. Thus, for medical students, like their patients, we posit that flourishing with adversity can occur and does so in the biopsychosocial context of human development, impacting health and well-being across the lifespan.

Based on our epidemiologic studies, we have established the potential importance of safe and secure relationships in the ability to flourish with adversity [18-20]. We have further posited that flourishing with adversity occurs as individuals develop relational capacities by experiencing relationships in which they feel safe and seen [21]. In LEC, our intention as faculty is to model the relational capacities we are trying to cultivate in the students. As medical students develop these capacities, they can enter into their relationships and experiences, inside and outside of work, in a way that helps them flourish, better discerning their own strengths, limitations and the kind of physician they wish to become—learning who they are and becoming who they are.

In LEC, we believe that the practice of safe, relationship-based reflection increases relational capacities through more than faculty modeling. The practice allows students to integrate experiences and processes (psychological and even physiological) that are often separated in the teaching and practice of medicine, such as the separation between feeling and thinking, past and present, personal and professional, nature and nurture, self and other, and healer and patient. Consistent with the goal of flourishing with adversity, the reflection in LEC is not just about managing stress and adversity but also about exploring the nature of call [22, 23] and the search for self-transcendent purpose that often brings students to the field of medicine. This search is often shaped by the opportunity for students to reflect on how their life experiences have shaped their talents and the meaning they might derive in using them in medicine. We focus on students' assets and positive experiences and not just on their limitations and distress. For some students, there is the opportunity to safely explore how certain adaptations to their own adverse life experiences [24] may also be the basis of certain strengths, such as empathy and attunement, which when accepted and managed can be gifts for patients and colleagues rather than personal liabilities.

Section S3. The Columbia-Bassett Program and the development of the Life Experiences Curriculum

We developed LEC within the Columbia-Bassett (CB) Program [25]. CB was developed by one of us (HW) and began in 2010 as a medical education track within Columbia University's Vagelos College of Physicians and Surgeons. Approximately 10 students admitted each year to the medical school are also admitted to the CB track through a separate application. A defining feature of the track is the longitudinal integrated curriculum during the major clinical year of training, when students complete their core clinical rotations. These rotations occur in a rural healthcare system in central New York State (Bassett Healthcare Network), where the students have the opportunity for longitudinal care experiences with patients and longitudinal learning experiences with their clinical preceptors. In this way, both receiving care and learning to give care occur in relationships over time. From its inception, CB had intentions that are reflected in LEC, such as recognizing the adversity inherent within healthcare and medical education, emphasizing the high prevalence and negative impact of adverse childhood experiences, prioritizing students' psychological safety, and cultivating students' self-awareness and self-acceptance.

The formal development of LEC began in December 2015 with a symposium at Bassett Medical Center (Cooperstown, NY) entitled, "Compassionate Healing of Root Causes." Thought leaders on the biopsychosocial impacts of trauma provided perspectives to CB clinical faculty

and clinical-year students about how best to teach medical students to care for people who might be impacted by traumatic life experiences. The invited presenters included Bessel van der Kolk, MD (developmental trauma), Sandra L. Bloom, MD (organizational trauma), Bruce S. McEwen, PhD (developmental neurobiology of stress), and Robert C. Whitaker, MD, MPH (developmental origins of flourishing). The symposium generated themes that have endured as core principles in LEC, such as the following: flourishing and adversity can coexist; positive and traumatic life experiences occur in the biopsychosocial context of human development and impact health and well-being across the lifespan; and trauma to individuals and organizations reduces psychological safety and that safety must be restored for healing. Beyond the discussions at the 2015 symposium, there was no formal needs assessment that informed the development of LEC. Although students participated in that symposium and have provided ongoing feedback during LEC implementation, they were not otherwise involved in the development of LEC.

In January 2018, three of us (RW, AH, and TDW) joined CB to develop a research program that would further inform the development of LEC by focusing on how relationships in healthcare, education, and human services can promote well-being by being more responsive to people's life experiences. The early work of that research program involved the evaluation of a six-session (15-hour) course, called Enhancing Trauma Awareness, using a randomized trial involving 96 early childhood education professionals [26, 27]. This course and others were developed by Lakeside Global Institute (2024) (North Wales, PA) for various professionals in education and human services.

Both the content and process of LEC were informed by these Lakeside courses and the almost 25 years of experience accumulated by Lakeside in developing and administering its trauma-training courses to those in various helping professions outside of healthcare. We selected content for LEC that we deemed most appropriate for medical students. We began with the seminal work of our 2015 seminar speakers on the topics of developmental trauma [28], organizational trauma [29], the developmental neurobiology of stress [30], and the developmental origins of flourishing [18]. We then examined the available literature at that time on trauma-informed medical education (for example: [31-33]). We also consulted best practices emerging from the work of the National Collaborative on Trauma-Informed Health Care Education and Research. In addition, we reviewed the literature on trauma-informed care (TIC) in relevant settings outside medical education (for example: [34, 35]). Finally, all three LEC co-leaders (RW, MO, MB) undertook further training in TIC. One of us (RW), a pediatrician, participated in a three-course certificate program (75 hours) through Lakeside Global Institute [36], and two of us (MO and MB), both psychiatrists, took a two-day course (16 hours) through McLean Hospital (Belmont, MA).

Since LEC implementation began, we have continued to monitor the emerging literature in trauma-informed medical education [37], including the development of competencies for students and faculty regarding TIC in undergraduate medical education [38]. Medical schools have developed trauma-informed medical education curricula that address topics such as the clinical skills of history-taking [39] and physical examination [40, 41]. These topics have not been our focus because they are addressed elsewhere in Columbia's medical school curriculum. Content of these emerging curricula have included topics we address in the LEC pre-clinical seminar sessions (**Manuscript Table 1**), such as the following: the Substance Abuse and Mental Health Services Administration's (2014) definition of trauma and the six-part approach to TIC,

adverse childhood experiences, biopsychosocial consequences of trauma, and application of these principles to clinical encounters [42, 43]. Other authors have also emphasized the importance of addressing students' psychological safety and acknowledging students' own traumatic experiences [31, 44], but they do not describe specific strategies.

Section S4. The rationale for prioritizing psychological safety in the Life Experiences Curriculum

The learning and clinical environments in medical education and healthcare can confront students with events and circumstances that are emotionally charged, evoke feelings of vulnerability and shame, and are sometimes even traumatic. Therefore, establishing psychological safety, the first principle of trauma-informed care [45], is the major priority of our process in LEC. Without psychologically safe spaces for students to reflect and express their thoughts and feelings, the natural tendency is to persist in defending against them. While this can initially be adaptive, particularly in unsafe environments, defenses may inhibit growth and integration and become maladaptive in the long run.

Safe spaces for reflection and sharing about the content of the LEC curriculum, clinical encounters, and the systems of medical education and healthcare can allow both students and faculty to integrate their past and ongoing experiences. Given the opportunity to reflect regularly throughout medical school within a caring community of classmates and teachers, in which psychological safety is established over time, students may be able to experience, tolerate, express, and learn from certain thoughts and feelings that might otherwise be suppressed. In addition, psychological safety optimizes openness to new experiences, including learning new material that may also be emotionally challenging and otherwise avoided. Safety enhances curiosity about the unknown and creativity around the fundamental challenge of flourishing in the face of ubiquitous adversity. By experiencing and naming the felt sense of safety, students are able to embody it and cultivate it in their relationships with their patients and colleagues.

Section S5. Future directions

As the scientific evidence increases about the nature, impacts, and treatment of traumatic life experiences, there will be increasing pressure to add content to trauma-informed medical education. It is our view that attention to process will remain more important than adding content. For example, we have much to learn about how best to increase students' awareness and compassion for their own life experiences and not just those in their care. Accordingly, we see the greatest future possibility for LEC in working with students to build a "communal and relational field of caring support" that yields "sustainable and inclusive compassion" [46 p1346]. This means that the community built in LEC can serve as a collective secure base to which its members can return when faced with adversity and that the community includes anyone wishing to receive care as a starting point for giving care. To be included means overcoming the challenge, faced by many physicians, of feeling worthy to receive care as well as give care. Our experience to date is that this may involve overcoming blocks to self-compassion and reflective functioning, some of which may be related to developmental trauma [47, 48]. While there is emerging evidence about approaches to overcoming such blocks in those with mental health disorders [49], it is not clear to us as yet if or how this evidence might be applied to medical students in the context of LEC [50]. Building communities of care in healthcare will require a

cultural shift away from caregiving as independent and individual to interdependent and collective. Moving away from our perceptions of the "solo self" [51 p24] and towards communities of care and support may be "the relational starting point" [46 p1347] for not only preventing burnout in medicine but for cultivating the ability to flourish with adversity.

Table S1. Proposed LEC evaluation outcome measures

Construct	Instrument	Sample Items
**Primary outcome: Relational capacity score[a]**		
Self-awareness: Mindfulness	Five Facet Mindfulness Questionnaire, Short Form (15-items, 5 subscales, 3 items each) (Baer et al., 2006; Baer et al., 2008; Gu et al., 2016) [52-54]	1. Observing: "I pay attention to sensations, such as the wind in my hair or sun on my face."<br>2. Describe: "Even when I'm feeling terribly upset I can find a way to put it into words."<br>3. Acting with awareness: "I find myself doing things without paying attention." (reverse-scored)<br>4. Non-judging: "I think some of my emotions are bad or inappropriate and I shouldn't feel them." (reverse-scored)<br>5. Non-reactivity: "When I have distressing thoughts or images, I "step back" and am aware of the thought or image without getting taken over by it."
Self-acceptance: Self-compassion	Self-Compassion Scale, Short Form (12-items, 6 subscales, 2 items each)<br>(Raes et al., 2011) [55]	1. Self-kindness: "When I'm going through a very hard time, I give myself the caring and tenderness I need."<br>2. Self-judgment: "I'm intolerant and impatient towards those aspects of my personality I don't like." (reverse-scored)<br>3. Common humanity: "When I feel inadequate in some way, I try to remind myself that feelings of inadequacy are shared by most people."<br>4. Isolation: "When I fail at something that's important to me, I tend to feel alone in my failure." (reverse-scored)<br>5. Mindfulness: "When something painful happens I try to take a balanced view of the situation."<br>6. Over-identified: "When I fail at something important to me I become consumed by feelings of inadequacy." (reverse-scored)
Other-awareness: Empathy	Interpersonal Reactivity Index (14-items, 2 subscales, 7 items each)<br>(Davis, 1980; Pulos et al., 2004) [56, 57]	1. Perspective-taking: "When I'm upset at someone, I usually try to 'put myself in their shoes' for a while."<br>2. Empathic concern: "I often have tender, concerned feelings for people less fortunate than me."

Construct	Instrument	Sample Items
Other-acceptance: Compassion	Compassion Scale (16-items, 4 subscales, 4 items each) (Pommier et al., 2020) [58]	1. Kindness: "If I see someone going through a difficult time, I try to be caring toward that person."<br>2. Common humanity: "I feel it's important to recognize that all people have weaknesses and no one's perfect."<br>3. Mindfulness: "I pay careful attention when other people talk to me about their troubles."<br>4. Indifference: "I think little about the concerns of others." (reverse-scored)
**Secondary outcome: Flourishing Score[b]**		
Purpose in life	Psychological Well-Being Scale (7-item subscale) (Ryff, 1989) [9]	"I have a sense of direction and purpose in life."
Self-acceptance	Psychological Well-Being Scale (7-item subscale) (Ryff, 1989) [9]	"In general, I feel confident and positive about myself."
Positive relations with others	Psychological Well-Being Scale (7-item subscale) (Ryff, 1989) [9]	"I enjoy personal and mutual conversations with family members and friends."
Personal growth	Psychological Well-Being Scale (7-item subscale) (Ryff, 1989) [9]	"I think it is important to have new experiences that challenge how you think about yourself and the world."
Environmental mastery	Psychological Well-Being Scale (7-item subscale) (Ryff, 1989) [9]	"I am quite good at managing the many responsibilities of my daily life."
Autonomy	Psychological Well-Being Scale (7-item subscale) (Ryff, 1989) [9]	"I judge myself by what I think is important, not by the values of what others think is important."

a. See [21] for method of constructing the relational capacity score from the 57 items across 4 instruments and 17 subscales.

b. See [18] for method of constructing the flourishing score from the 42 items across 6 subscales.

References

1. **Berger JT.** Moral distress in medical education and training. J Gen Intern Med. 2014;29(2):395-8. DOI: https://doi.org/ 10.1007/s11606-013-2665-0

2. **Thibodeau PS, Nash A, Greenfield JC, Bellamy JL.** The association of moral injury and healthcare clinicians' wellbeing: a systematic review. Int J Env Res Public Health. 2023;20(13):6300. DOI: https://doi.org/10.3390/ijerph20136300

3. **Porges SW.** Polyvagal theory: a science of safety. Front Integr Neurosci. 2022;16. DOI: https://doi.org/10.3389/fnint.2022.871227

4. **Rotenstein LS, Ramos MA, Torre M, Segal JB, Peluso MJ, Guille C, et al.** Prevalence of depression, depressive symptoms, and suicidal ideation among medical students: a systematic review and meta-analysis. JAMA. 2016;316(21):2214-36. DOI: https://doi.org/10.1001/jama.2016.17324

5. **Keyes CL.** Mental illness and/or mental health? Investigating axioms of the complete state model of health. J Consult Clin Psychol. 2005;73(3):539-48. DOI: https://doi.org/10.1037/0022-006X.73.3.539

6. **Arnett JJ, Žukauskienė R, Sugimura K.** The new life stage of emerging adulthood at ages 18–29 years: implications for mental health. Lancet Psychiatry. 2014;1(7):569-76.

7. **Huta V, Waterman AS.** Eudaimonia and its distinction from hedonia: developing a classification and terminology for understanding conceptual and operational definitions. J Happiness Stud. 2014;15(6):1425-56. DOI: https://doi.org/10.1007/s10902-013- 9485-0

8. **Ryan RM, Deci EL.** On happiness and human potentials: a review of research on hedonic and eudaimonic well-being. Annu Rev Psychol. 2001;52(1):141-66. DOI: https://doi.org/10.1146/annurev.psych.52.1.141

9. **Ryff CD.** Happiness is everything, or is it? Explorations on the meaning of psychological well-being. J Pers Soc Psychol. 1989;57(6):1069-81. DOI: https://doi.org/10.1037/0022-3514.57.6.1069

10. **Ryff CD, Singer BH.** Know thyself and become what you are: a eudaimonic approach to psychological well-being. J Happiness Stud. 2008;9:13-39. DOI: https://doi.org/10.1007/s10902-006-9019-0

11. **Ryff CD.** Psychological well-being revisited: advances in the science and practice of eudaimonia. Psychother Psychosom. 2014;83(1):10-28. DOI: https://doi.org/10.1159/000353263

12. **Ryff CD, Singer B.** Flourishing under fire: resilience as a prototype of challenged thriving. In: Keyes CLM & Haidt J (Eds.) Flourishing: positive psychology and the life well-lived. Washington, D.C: American Psychological Association; 2003. pp. 15-36. DOI: https://doi.org/10.1037/10594-001

13. **Masten AS.** Resilience theory and research on children and families: past, present, and promise. J Fam Theory Rev. 2018;10(1):12-31. DOI: https://doi.org/10.1111/jftr.12255

14. **Tedeschi RG, Calhoun LG.** Posttraumatic growth: conceptual foundations and empirical evidence. Psychol Inq. 2004;15(1):1-18. DOI: http://www.jstor.org.tc.idm.oclc.org/stable/20447194

15. **Dyrbye LN, Satele D, West CP.** Association of characteristics of the learning environment and US medical student burnout, empathy, and career regret. JAMA Netw Open. 2021;4(8):e2119110. DOI: https://doi.org/10.1001/jamanetworkopen.2021.19110

16. **Talbot SG, Dean W.** Physicians aren't 'burning out.' They're suffering from moral injury. Stat. (26 July 2018). https://www.statnews.com/2018/07/26/physicians-not-burning-out-they-are-suffering-moral-injury/ (accessed 13 March 2024).

17. **Wiggleton C, Petrusa E, Loomis K, Tarpley J, Tarpley M, O'Gorman ML, et al.** Medical students' experiences of moral distress: development of a web-based survey. Acad Med. 2010;85(1):111-7.

18. **Whitaker RC, Dearth-Wesley T, Herman AN.** Childhood family connection and adult flourishing: associations across levels of childhood adversity. Acad Pediatr. 2021. DOI: https://doi.org/10.1016/j.acap.2021.03.002

19. **Whitaker RC, Dearth-Wesley T, Herman AN, Nagel KE, Smith HG, Weil HFC.** Association of childhood family connection with flourishing in young adulthood among those with type 1 diabetes. JAMA Netw Open. 2020;3(3):e200427. DOI: https://doi.org/10.1001/jamanetworkopen.2020.0427

20. **Whitaker RC, Dearth-Wesley T, Herman AN, Benz TL, Saint-Hilaire SA, Strup DD.** The association between teacher connection and flourishing among early adolescents in 25 countries. J Early Adolesc. 2023. DOI: https://doi.org/10.1177/02724316231190828

21. **Whitaker RC, Herman AN, Dearth-Wesley T.** Relational health as a pathway from trauma to flourishing in school communities. J Sch Health. 2023;93(7):628-37. DOI: https://doi.org/10.1111/josh.13282

22. **Yoon JD, Daley BM, Curlin FA.** The association between a sense of calling and physician well-being: a national study of primary care physicians and psychiatrists. Acad Psychiatry. 2017;41:167-73. DOI: https://doi.org/10.1007/s40596-016-0487-1

23. **Dik BJ, Duffy RD.** Calling and vocation at work: definitions and prospects for research and practice. Couns Psychol. 2009;37(3):424-50. DOI: https://doi.org/10.1177/0011000008316430

24. **Sciolla AF, Wilkes MS, Griffin EJ.** Adverse childhood experiences in medical students: implications for wellness. Acad Psychiatry. 2019;43:369-74. DOI: https://doi.org/10.1007/s40596-019-01047-5

25. **Columbia University Irving Medical Center.** Columbia-Bassett program. https://www.vagelos.columbia.edu/education/academic-programs/md-program/columbia-bassett-program (accessed 13 March 2024).

26. **Herman AN, Whitaker RC.** Reconciling mixed messages from mixed methods: a randomized trial of a professional development course to increase trauma-informed care. Child Abuse Negl. 2020;101:104349. DOI: https://doi.org/10.1016/j.chiabu.2019.104349

27. **Whitaker RC, Herman AN, Dearth-Wesley T, Smith HG, Burnim SB, Myers EL, et al.** Effect of a trauma-awareness course on teachers' perceptions of conflict with preschool-aged children from low-income urban households: a cluster randomized clinical trial. JAMA Netw Open. 2019;2(4):e193193. DOI: https://doi.org/10.1001/jamanetworkopen.2019.3193

28. **van der Kolk BA.** The body keeps the score: brain, mind, and body in the healing of trauma. New York, NY: Viking; 2014.

29. **Bloom SL.** Creating sanctuary: toward the evolution of sane societies, revised ed. New York, NY: Routledge; 2013.

30. **McEwen BS.** Protective and damaging effects of stress mediators. New Engl J Med. 1998;338(3):171-9. DOI: https://doi.org/10.1056/NEJM199801153380307

31. **Brown T, Berman S, McDaniel K, Radford C, Mehta P, Potter J, et al.** Trauma-informed medical education (TIME): advancing curricular content and educational context. Acad Med. 2021;96(5):661-7. DOI: https://doi.org/10.1097/ACM.0000000000003587

32. **Lewis-O'Connor A, Warren A, Lee JV, Levy-Carrick N, Grossman S, Chadwick M, et al.** The state of the science on trauma inquiry. Womens Health. 2019;15:1745506519861234. DOI: https://doi.org/ 10.1177/1745506519861234

33. **Machtinger EL, Davis KB, Kimberg LS, Khanna N, Cuca YP, Dawson-Rose C, et al.** From treatment to healing: inquiry and response to recent and past trauma in adult health care. Womens Health Issues. 2019;29(2):97-102. DOI: https://doi.org/10.1016/j.whi.2018.11.003

34. **Purtle J.** Systematic review of evaluations of trauma-informed organizational interventions that include staff trainings. Trauma Violence Abuse. 2020;21(4):725-40. DOI: https://doi.org//10.1177/1524838018791304

35. **Blodgett C, Dorado J.** A selected review of trauma-informed school practice and alignment with educational practice. San Francisco, CA: CLEAR Trauma Center, University of California San Francisco; 2016. Retrieved from https://s3.wp.wsu.edu/uploads/sites/2101/2019/12/Selected-Review-of-Trauma-Informed-School-Practice-2016.pdf.

36. **Lakeside.** Intensive Courses. https://lakesidelink.com/training/professional-development/intensive-courses/ (accessed 13 March 2024).

37. **Burns CJ, Borah L, Terrell SM, James LN, Erkkinen E, Owens L.** Trauma-informed care curricula for the health professions: a scoping review of best practices for design, implementation, and evaluation. Acad Med. 2023;98:401-9. DOI: https://doi.org/10.1097/ACM.0000000000005046

38. **Berman S, Brown T, Mizelle C, Diep T, Gerber MR, Jelley M, et al.** Roadmap for trauma-informed medical education: Introducing an essential competency set. Acad Med. 2023;98(8):882-8. DOI: https://doi.org/ 10.1097/ACM.0000000000005196

39. **Brown T, Mehta PK, Berman S, McDaniel K, Radford C, Lewis-O'Connor A, et al.** A trauma-informed approach to the medical history: teaching trauma-informed communication skills to first-year medical and dental students. MedEdPORTAL. 2021;17:11160. DOI: https://doi.org/10.15766/mep_2374-8265.11160

40. **Elisseou S, Adams E, Adler M.** A trauma-informed approach to peer physical examination. MedEdPORTAL. 2022;18:11273. DOI: https://doi.org/10.15766/mep_2374-8265.11273

41. **Elisseou S, Puranam S, Nandi M.** A novel, trauma-informed physical examination curriculum for first-year medical students. MedEdPORTAL. 2019;15:10799. DOI: https://doi.org/10.15766/mep_2374-8265.10799

42. **Chokshi B, Walsh K, Dooley D, Falusi O, Deyton L, Beers L.** Teaching trauma-informed care: a symposium for medical students. MedEdPORTAL. 2020;16:11061. DOI: https://doi.org/10.15766/mep_2374-8265.11061

43. **Pletcher BA, O'Connor M, Swift-Taylor ME, DallaPiazza M.** Adverse childhood experiences: a case-based workshop introducing medical students to trauma-informed care. MedEdPORTAL. 2019;15:10803. DOI: https://doi.org/10.15766/mep_2374-8265.10803

44. **Grossman S, Berman S, Brown T, Lewis-O'Connor A, McDaniel K, Mehta P, et al.** Implementing a trauma-informed care curriculum in undergraduate medical education. In: Douglass LL, Threlkeld A, & Merriweather LR (Eds.) Trauma in adult and higher education: conversations and critical reflections. Charlotte, NC: Information Age Publishing, Inc.; 2021. pp. 187-204.

45. **Substance Abuse and Mental Health Services Administration**. SAMHSA's concept of trauma and guidance for a trauma-informed approach. HHS Publication No. (SMA) 14-4884. Rockville, MD: Substance Abuse and Mental Health Services Administration; 2014. Retrieved from https://store.samhsa.gov/system/files/sma14-4884.pdf.

46. **Condon P, Makransky J.** Recovering the relational starting point of compassion training: a foundation for sustainable and inclusive care. Perspect Psychol Sci. 2020;15(6):1346-62. DOI: https://doi.org/10.1177/1745691620922200

47. **Gilbert P, McEwan K, Matos M, Rivis A.** Fears of compassion: development of three self-report measures. Psychol Psychother. 2011;84(3):239-55. DOI: https://doi.org/10.1348/147608310X526511

48. **Fonagy P, Target M.** Attachment and reflective function: their role in self-organization. Dev Psychopathol. 1997;9(4):679-700. DOI: https://doi.org/10.1017/S0954579497001399

49. **Gilbert P.** The origins and nature of compassion focused therapy. Br J Clin Psychol. 2014;53(1):6-41. DOI: https://doi.org/10.1111/bjc.12043

50. **Janes E, Gibbons C, Gyane S, Irons C, Wilson M.** Experiences of a newly developed brief compassion-focused intervention for trainee mental health professionals – a qualitative study. SSM - Mental Health. 2024;5:100288. DOI: https://doi.org/https://doi.org/10.1016/j.ssmmh.2023.100288

51. **Siegel D.** IntraConnected: mwe (me + we) as the integration of self, identity, and belonging. W.W. Norton; 2022.

52. **Baer RA, Smith GT, Hopkins J, Krietemeyer J, Toney L.** Using self-report assessment methods to explore facets of mindfulness. Assessment. 2006;13(1):27-45. DOI: https://doi.org/10.1177/1073191105283504

53. **Baer RA, Smith GT, Lykins E, Button D, Krietemeyer J, Sauer S, et al.** Construct validity of the five facet mindfulness questionnaire in meditating and nonmeditating samples. Assessment. 2008;15(3):329-42. DOI: https://doi.org/10.1177/1073191107313003

54. **Gu J, Strauss C, Crane C, Barnhofer T, Karl A, Cavanagh K, et al.** Examining the factor structure of the 39-item and 15-item versions of the five facet mindfulness questionnaire before and after mindfulness-based cognitive therapy for people with recurrent depression. Psychol Assess. 2016;28(7):791. DOI: https://doi.org/10.1037/pas0000263

55. **Raes F, Pommier E, Neff KD, Van Gucht D.** Construction and factorial validation of a short form of the self-compassion scale. Clin Psychol Psychother. 2011;18(3):250-5. DOI: https://doi.org/10.1002/cpp.702

56. **Davis MH.** A multidimensional approach to individual differences in empathy. JSAS Catalog of Selected Documents in Psychology. 1980;10:85.

57. **Pulos S, Elison J, Lennon R.** The hierarchical structure of the interpersonal reactivity index. Soc Behav Pers. 2004;32(4):355-9. DOI: https://doi.org/10.2224/sbp.2004.32.4.355

58. **Pommier E, Neff KD, Tóth-Király I.** The development and validation of the compassion scale. Assessment. 2020;27(1):21-39. DOI: https://doi.org/10.1177/1073191119874108
